# Involvement of PrP^C^ in kainate-induced excitotoxicity in several mouse strains

**DOI:** 10.1038/srep11971

**Published:** 2015-07-09

**Authors:** Patricia Carulla, Franc Llorens, Andreu Matamoros-Angles, Patricia Aguilar-Calvo, Juan Carlos Espinosa, Rosalina Gavín, Isidre Ferrer, Giuseppe Legname, Juan Maria Torres, José A. del Río

**Affiliations:** 1 Molecular and Cellular Neurobiotechnology, Institute of Bioengineering of Catalonia (IBEC), Parc Científic de Barcelona, Barcelona, Spain; 2 Department of Cell Biology, Universitat de Barcelona, Barcelona, Spain; 3 Centro de Investigación Biomédica en Red sobre Enfermedades Neurodegenerativas (CIBERNED), Barcelona, Spain; 4 German Center for Neurodegenerative Diseases (DZNE), Robert-Koch Str. 40, 37075, Göttingen, Germany; 5 Centro de Investigación en Sanidad Animal (CISA-INIA), Valdeolmos, Madrid, Spain; 6 Institut de Neuropatologia, IDIBELL-Hospital Universitari de Bellvitge, Hospitalet de Llobregat, Barcelona, Spain; 7 Centro de Investigación Biomédica en Red de Enfermedades Neurodegenerativas (CIBERNED), Barcelona, Spain; 8 Laboratory of Prion Biology, Department of Neuroscience, Scuola Internazionale Superiore di Studi Avanzati (SISSA), Trieste, Italy

## Abstract

The cellular prion protein (PrP^C^) has been associated with a plethora of cellular functions ranging from cell cycle to neuroprotection. Mice lacking PrP^C^ show an increased susceptibility to epileptic seizures; the protein, then, is neuroprotective. However, lack of experimental reproducibility has led to considering the possibility that other factors besides PrP^C^ deletion, such as the genetic background of mice or the presence of so-called “*Prnp* flanking genes”, might contribute to the reported susceptibility. Here, we performed a comparative analysis of seizure-susceptibility using characterized *Prnp*^+/+^ and *Prnp*^0/0^ mice of B6129, B6.129, 129/Ola or FVB/N genetic backgrounds. Our study indicates that PrP^C^ plays a role in neuroprotection in KA-treated cells and mice. For this function, PrP^C^ should contain the aa32–93 region and needs to be linked to the membrane. In addition, some unidentified “*Prnp*-flanking genes” play a role parallel to PrP^C^ in the KA-mediated responses in B6129 and B6.129 *Prnp*^0/0^ mice.

Although the role of the cellular form of the prion protein (PrP^C^) in living organisms has been intensively studied, a clear consensus concerning the physiological functions of this protein is still elusive and controversial. To date, existing evidence implicates PrP^C^ in numerous distinct cellular processes, including cell proliferation and differentiation^1^,^2^, copper homeostasis^3^,^4^, oxidative stress^5^ and cell signaling^6^,^7^, among others.

The generation of different transgenic *Prnp*^0/0^ mice (mixed B6129 *Prnp*^Zrchl/Zrchl^ or co-isogenic 129/Ola *Prnp*^Edbg/Edbg^ backgrounds) in the early 1990s did not reveal any relevant phenotypic alteration of the mutant mice^8^,^9^. However, subsequent studies identified an abundance of phenotypic alterations (e.g.,^10^), including depressive-like behaviour^11^, cognitive deficits^12^, age-dependent behavioural abnormalities^13^, altered olfaction^14^, peripheral myelin deficits^15^, altered circadian rhythms^16^ and an increased susceptibility to oxidative stress^5^ and glutamate excitotoxicity^17^,^18^,^19^. Indeed, different laboratories have described in these strains and in congenic B6.129 *Prnp*^Zrchl/Zrchl^ (B6129 mice backcrossed with C57BL/6 for several generations) an enhanced sensitivity to seizures after the administration of epileptogenic drugs such as kainic acid (KA), N-methyl-d-aspartic acid (NMDA), pilocarpine and pentylenetetrazol (PTZ), suggesting a neuroprotective role of the protein against excitotoxic insults (e.g.,^17^,^18^,^19^,^20^,^21^). However, some studies suggest that PrP^C^ is not involved in KA-mediated excitotoxicity and that the observed differences are likely to be associated with the genetic background of the mice used in the experiments^22^,^23^,^24^. In fact, it was described some years ago how different mouse strains exhibit different vulnerability to the administration of glutamate and other seizure-inducing drugs, which correlates with differences in axonal sprouting and cell death in the hippocampal region (e.g.,^25^,^26^). For example, the hippocampus of the C57BL/6 background mouse, in contrast to FVB/N, is more resistant to the excitotoxic effects of KA^25^,^27^,^28^,^29^. Thus, these genetic differences dilute the specific participation of PrP^C^ in KA-mediated cell death. However, these genomic influences do not *per se* explain the neuroprotective properties of PrP^C^ observed in KA-treated neuroblastoma cell lines carrying different dosages of the *Prnp* gene (see below).

In addition to these genetic differences, a number of *Prnp*-flanking genes associated with the 129 genotype in B6129 *Prnp*^Zrchl/Zrchl^ mixed mice^30^ have recently been described. These genes are retained in *Prnp*^0/0^ progeny of congenic B6.129 *Prnp*^Zrchl/Zrchl^ after numerous (>10) crosses^30^,^31^ with C57BL/6 mice. SNP analysis of the backcrossed mice indicated that after 5 to 6 rounds the amount of C57BL/6-associated SNP increased from ≈60 up to ≈93%. Thus, B6.129 *Prnp*^Zrchl/Zrchl^ wild type and mutant mice may still differ at these additional polymorphic loci associated with *Prnp*^32^,^33^. One of these loci, the signal regulatory protein alpha (SIRPα) has recently been described as responsible for a previously PrP^C^-associated phagocytic function in macrophages^33^. More relevantly, overexpressing mice (Tg20) generated in mixed B6129 *Prnp*^Zrchl/Zrchl^ congenic mouse carried several copies of the polymorphic loci, since in most cases they are also crossed with previously backcrossed B6.129 *Prnp*^Zrchl/Zrchl^ mice^30^. In this scenario, it is reasonable to consider that the data obtained using *Prnp*^0/0^ or Tg20 (backcrossed or not with C57BL/6) in electrophysiological studies may yield conflicting results^24^,^34^,^35^,^36^. Indeed, lack of PrP^C^ has been associated with long-term potentiation (LTP), GABA-mediated fast after hyperpolarization (AHP) and paired pulse facilitation (PPF) abnormalities in mutant mice (e.g.,^37^,^38^,^39^,^40^). However, regulatory participation of PrP^C^ in neurotransmission and neuroprotection and in other cellular functions has also been shown with acute modulation of *Prnp* expression in neural cell lines and in other organisms (e.g., zebrafish) as models, to avoid the above-mentioned genetic problems of mice. For example, neuroblastoma (N2a) cells with reduced *Prnp* expression are more susceptible to KA in contrast to non-modified cells^18^,^20^. Functions of PrP^C^ in neurotransmission are also reinforced since i) PrP^C^ seems to be located in the synaptic cleft^20^,^41^,^42^,^43^; ii) several synaptic proteins (e.g., synaptophysin, synapsin Ib)^44^,^45^, glutamate receptor subunits (e.g., GluR6/7, NR2D, GluR1/2, mGluR1/5)^19^,^20^,^46^,^47^ and ion channels^36^ have been identified as interacting partners of PrP^C^; iii) PrP^C^ regulates GluR6/7-mediated signalling through the modulation of the formation of the GluR6/7-PSD-95-MLK3 trimeric complex, which triggers the activation of the JNK3 apoptotic pathway in the hippocampus in response to KA administration^20^; and iv) it modulates the NMDA receptor^48^.

Due to these conflicting results, we aimed to clarify the participation of PrP^C^ in KA-mediated excitotoxicity *in vivo*, using B6129 *Prnp*^Zrchl/Zrchl^, B6.129 *Prnp*^Zrchl/Zrchl^, 129/Ola *Prnp*^Edbg/Edbg^ and FVB *Prnp*^0/0^ and *Prnp*^+/+^ mice, and *in vitro* using N2a cells. In addition, we wished to explore whether the expression of truncated forms of PrP^C^ (ΔF35 or ΔC4) lacking 32–134 and 32–93 residues of PrP^C^ might increase the neurotoxic effects.

Our results indicate that FVB/N *Prnp*^0/0^ mice are not suitable for the *in vivo* model of KA-mediated excitotoxicity due to the intrinsic sensitivity to KA of FVB/N mice that hides clear differences^49^,^50^. More relevantly, both B6129 *Prnp*^Zrchl/Zrchl^ and co-isogenic 129/Ola *Prnp*^Edbg/Edbg^ mice display more severe epileptic episodes and neurotoxic brain damage than their corresponding wild-type controls. Backcrossed B6.129 *Prnp*^Zrchl/Zrchl^ mice (with ≈93% of C57BL/6 SNPs) displayed lower numbers of seizures compared to B6129, indicating additional participation of *Prnp*-linked genes in KA-mediated effects in B6129 *Prnp*^Zrchl/Zrchl^ mice. However, our data using co-isogenic 129/Ola *Prnp*^Edbg/Edbg^ (100% 129 genotype) and cell lines reinforce the notion that PrP^C^ is neuroprotective in KA treatment. In addition, the expression of ΔF35 and ΔC4 in a B6129 *Prnp*^Zrchl/Zrchl^ background increased hippocampal cell death, especially in the CA3 region after KA injections. These *in vivo* data were also corroborated using N2a cells transiently expressing N-terminal truncated forms of PrP^C^. Lastly, our *in vitro* experiments also suggest that to be neuroprotective, PrP^C^ should be bound to the plasma membrane by means of the GPI moiety.

## Results

### KA-induced seizures in several strains of wild-type and PrP^C^-null mice

**Figure 1:**
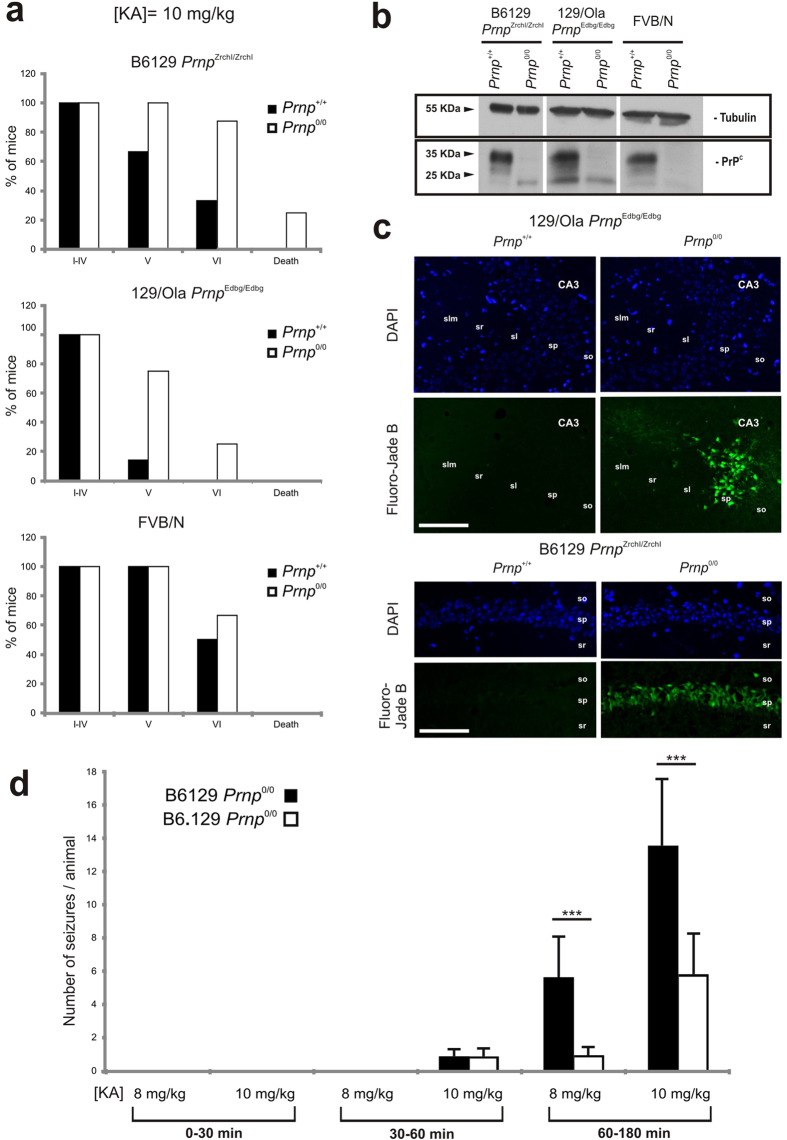
Comparison of KA-induced seizure profile in *Prnp*^*+/+*^and *Prnp*^0/0^ mice on B6129, B6.129, 129/Ola or FVB/N genetic backgrounds. (**a**) Percentage of mice reaching seizure stages I to VI and KA-induced mortality. Adult animals were subjected to a multiple KA-injection protocol (10 mg/kg b.w.) and epileptic responses were analysed during 4 hours after the first KA injection (see Methods for details).(**b**) Example of the western blot detection of PrP^C^ (6H4) expression in brain extracts obtained from B6129 *Prnp*^Zrchl/Zrchl^ *Prnp*^+/+^, B6129 *Prnp*^Zrchl/Zrchl^ *Prnp*^0/0^, 129/Ola *Prnp*^Edbg/Edbg^ *Prnp*^+/+^, 129/Ola *Prnp*^Edbg/Edbg^ *Prnp*^0/0^, FVB/N *Prnp*^+/+^ and FVB/N *Prnp*^0/0^ mice. Tubulin was used as a loading control. (**c**) Photomicrographs showing the pattern of neurodegeneration (Fluoro-Jade B staining) and DAPI staining of hippocampus in B6129 *Prnp*^Zrchl/Zrchl^ *Prnp*^+/+^, B6129 *Prnp*^Zrchl/Zrchl^ *Prnp*^0/0^, 129/Ola *Prnp*^Edbg/Edbg^ *Prnp*^+/+^, 129/Ola *Prnp*^Edbg/Edbg^ *Prnp*^0/0^, 24 hours after KA treatment. Dying cells are mainly located in the pyramidal cell layer of CA1 and CA3 regions of B6129 *Prnp*^Zrchl/Zrchl^ *Prnp*^0/0^ and 129/Ola *Prnp*^Edbg/Edbg^ *Prnp*^0/0^ mice respectively. (**d**) Time course of stage VI seizure during the first 30 min, from 30–60 min and from 60–180 minutes, of adult B6129 *Prnp*^Zrchl/Zrchl^ *Prnp*^0/0^ vs B6.129 *Prnp*^Zrchl/Zrchl^ *Prnp*^0/0^. Data are presented as the mean ± S.E.M. of the number of seizures/number of animals. Note the lower levels of seizures in parallel with decreased 129 associated genes in B6.129 *Prnp*^Zrchl/Zrchl^ *Prnp*^0/0^ mice. Abbreviations: sr, *stratum radiatum*; sl, *stratum lucidum*; slm, stratum lacunosum-moleculare; sp, *stratum pyramidale*; so, *stratum oriens*. Scale bar represents 200 μm. Asterisks indicate statistical significance (***P < 0.01, Mann-Whitney *U* test).

A gene-targeting strategy to generate null mutations in mice is a powerful research tool to reveal *in vivo* the function of a single protein, as derived phenotypic alterations are usually attributed to the deleted gene. Nevertheless, behavioural alterations observed in null-mutant mice could result in some cases from the genetic background (see introduction, see also^26^,^49^,^50^). In order to determine a possible influence of non-*Prnp* genes in the susceptibility to KA-induced seizures, we compared the epileptic response in B6129 (n = 20), 129/Ola *Prnp*^Edbg/Edbg^ (n = 9) and FVB/N *Prnp*^0/0^ (n = 7) and wild type mice (B6129, n = 16; 129/Ola *Prnp*^Edbg/Edbg^ (n = 11); FVB/N (n = 8) (Fig. 1a and Supplementary Tables 1–6). A multiple administration protocol, consisting of three intraperitoneal injections of KA (10 mg/kg body weight) at 30 min intervals, was used in this experiment. Seizure intensity was analysed during the 4 hours after the first KA injection and scored as indicated in Methods. For an easier comparative analysis, data from grades I to IV were grouped together.

After the behavioural study, mice were numbered and kept in separate boxes until histological studies. Percentages of the different strains of mice reaching each stage were represented (Fig. 1a). As indicated (Fig. 1a and Supplementary Tables 1–6), all mice achieved stages I–IV, developing hypoactivity and immobility shortly after the first injection. When comparing wild type *vs Prnp*-null mice, significant differences were observed in the percentage of mice reaching stages V and VI, with the B6129 *Prnp*^Zrchl/Zrchl^ and 129/Ola *Prnp*^Edbg/Edbg^ much more susceptible to seizures than their respective *Prnp*^+/+^ controls (Fig. 1a and Supplementary Movies 1 and 2). In both groups, a high percentage of *Prnp*-null mice (100% and 75% respectively) developed loss of balance control and intermittent whole-body convulsions. More severe seizure activity, consisting of continuous seizures and/or ‘popcorn’ bouncing behaviour (stage VI) was also reported, leading to the death of 20% of the B6129 *Prnp*^Zrchl/Zrchl^ mice tested. In contrast, no B6129 *Prnp*^+/+^ or 129/Ola *Prnp*^+/+^ mice reached stage VI or died during experiments.

Distinct results were obtained when analysing FVB/N mice, since no significant differences were observed between FVB/N *Prnp*^+/+^ and FVB/N *Prnp*^0/0^ animals, and only a 9% difference was observed between them (Fig. 1a and Supplementary Movie 3). FVB/N *Prnp*^+/+^ showed a high epileptic response at 10 mg/kg b.w of KA (≈50%), which was not observed in B6129 *Prnp*^+/+^ (≈30%) and 129/Ola *Prnp*^+/+^ (0%); it occurred in all described behavioural stages, but without any reported deaths. Although other unknown factors might be involved, such phenotypical differences between *Prnp*^+/+^ mouse strains could be related to small differences in PrP^C^ expression levels, since our western blot analysis revealed lower PrP^C^ expression in FVB/N *Prnp*^+/+^ (0.623 ± 0.04) compared to 129/Ola *Prnp*^+/+^ (0.92 ± 0.038) mice (*t* = 4.21, mean diff. = 0.30; 95% Cl of diff. = 0.66 to 0.54) but not between B6129 *Prnp*^+/+^ (0.84 ± 0.063) and FVB/N *Prnp*^+/+^ mice (*t* = 3,104, mean diff. = 0.22; 95% Cl of diff. = 0.−0.013 to 0.459), or between B6129 *Prnp*^+/+^ and 129/Ola *Prnp*^+/+^ (*t* = 1.112, mean diff. = −0.08; 95% Cl of diff. = −0.3166 to 0.1566) (Fig. 1b and Supplementary Fig. 1). It has been previously reported that excitotoxic damage derived from KA administration primarily affects pyramidal cells from CA1 and CA3 regions of the hippocampus. To corroborate that the increased cell death is associated with PrP^C^ deletion, we focused on B6129 *Prnp*^0/0^ and 129/Ola *Prnp*^0/0^ mice. We obtained coronal brain sections (dorsal hippocampus) from these mice 24 hours after KA treatment and performed Fluoro-Jade B staining to determine the associated neurodegeneration in the hippocampus. Wild type mice were processed in parallel (Fig. 1c). B6129- *Prnp*^0/0^ mice displayed much more pronounced pyramidal cell damage, with higher numbers in CA1 than in CA3, than wild-type controls, in which no Fluoro-Jade B positive cells were observed. PrP^C^-dependent differences were also detected between 129/Ola- *Prnp*^0/0^ and 129/Ola-*Prnp*^+/+^ although cell death appeared to be restricted mostly to the CA3 region (Fig. 1c).

Taken together, these results support the notion that the genetic background of the different *Prnp*^0/0^ mice plays a role in KA susceptibility^22^,^23^, with the FVB/N background more prone to seizures than 129/Ola or the mixed genetic background B6129^26^,^49^. The susceptibility of the FVB background^49^,^50^ probably masks participation of PrP^C^ at this specific KA-dosage.

More relevantly, the present results demonstrate a role of PrP^C^ in neuroprotection, since the phenotypic differences observed between *Prnp*^0/0^ and *Prnp*^+/+^ mice in B6129 *Prnp*^Zrchl/Zrchl^ (Supplementary Movie 1) are also reproduced in co-isogenic 129/Ola *Prnp*^Edbg/Edbg^ mice (Supplementary Movie 2).

### Decreased seizures in B6.129 *Prnp* ^Zrchl/Zrchl^ compared to B6129 *Prnp* ^Zrchl/Zrchl^ mice

In a second set of experiments we used B6.129 *Prnp*^Zrchl/Zrchl^ mice generated after crossing the original B6129 *Prnp*^Zrchl/Zrchl^ mice with C57BL/6 mice over several generations. In these experiments, B6129 *Prnp*^Zrchl/Zrchl^ mice were purchased directly from EMMA (Monterotondo, Italy) and they carried approximately ≈64% of C57BL/6 microsatellite markers (Charles River Laboratories) with ≈46% of non-C57BL/6 markers (129 in origin). We backcrossed these *Prnp*^0/0^ mice with C57BL/6 mice over several generations (8–10) to reduce the non-C57BL/6 microsatellite markers to ≈6.5–7%. The presence of C57BL/6 or 129 markers in all phenotypes used in the present study was determined by the genetic testing service at Charles River Laboratories. In the test, 110 microsatellite markers at approximately 15 cM intervals were analyzed, spreading across the 19 autosomes and the X chromosome, which distinguishes among 129 microsatellite markers ranging from 92 to 94% of C57BL/6 (in B6.129 *Prnp*^Zrchl/Zrchl^ mice). Thus B6129 and B6.129 *Prnp*^Zrchl/Zrchl^ mice were treated with 8 mg/kg b.w. (B6129 n = 8 and B6.129 *Prnp*^Zrchl/Zrchl^ n = 6) or 10 mg/kg b.w (B6129 n = 8 and B6.129 *Prnp*^Zrchl/Zrchl^ n = 9) of KA and the number of seizures was determined during the first 30 minutes, from 30 to 60 minutes and from 60 to 180 minutes postinjection. In this experiment each mouse received a KA injection every 30 minutes as above. Data revealed a relevant number of seizures in B6129 *Prnp*^Zrchl/Zrchl^ *Prnp*^0/0^ mice compared with B6.129 *Prnp*^Zrchl/Zrchl^ *Prnp*^0/0^ (KA 8 mg/kg *P* = 0.0021; KA 10 mg/kg *P* = 0.0086; Mann-Whitney *U* test confidence interval (CI) = 95% (Fig. 1d and Supplementary Movie 1). In addition, the differences between the numbers of seizures can be observed in both KA concentrations (8 and 10 mg/kg b.w). These data indicate that although PrP^C^ plays a role in the increased susceptibility to KA as demonstrated above, the 129 associated genes also play a role in the observed results.

### Increased susceptibility in B6.129-*Prnp* ^0/0^ and 129/Ola- *Prnp* ^0/0^ correlates with enhanced cell death, inflammatory markers and astrogliosis

**Figure 2:**
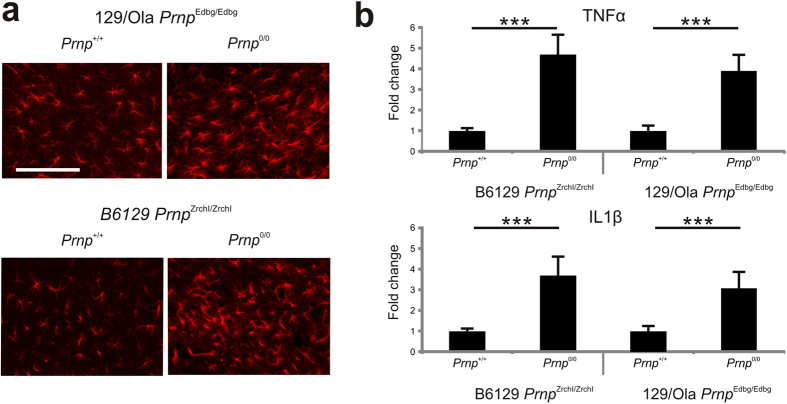
Increased astrogliosis, TNFα and IL1β expression in the hippocampus of B6129 *Prnp*^Zrchl/Zrchl^ *Prnp*^0/0^ and 129/Ola *Prnp*^Edbg/Edbg^ *Prnp*^0/0^. (**a**) Examples of GFAP-positive immunoreactive cells in the *stratum radiatum* of the hippocampal CA1-3, 24 hours after KA administration in hippocampal coronal sections of B6129 *Prnp*^Zrchl/Zrchl^ *Prnp*^+/+^, B6129 *Prnp*^Zrchl/Zrchl^ *Prnp*^0/0^, 129/Ola *Prnp*^Edbg/Edbg^ *Prnp*^+/+^, 129/Ola *Prnp*^Edbg/Edbg^ *Prnp*^0/0^. Both *Prnp*-null mice show greater numbers of reactive astroglia than their wild-type controls. (**b**) RT-qPCR of TNFα and IL1β mRNA levels from hippocampal RNA extracts obtained from B6129 *Prnp*^Zrchl/Zrchl^ *Prnp*^+/+^, B6129 *Prnp*^Zrchl/Zrchl^ *Prnp*^0/0^, 129/Ola *Prnp*^Edbg/Edbg^ *Prnp*^+/+^, 129/Ola *Prnp*^Edbg/Edbg^ *Prnp*^0/0^ mice 6 hours after the last KA treatment (at 60 minutes). Plotted data (mean ± S.E.M.) were obtained from three independent experiments and represented as mean fold change induction. Abbreviations are as in Fig. 1. Scale bars represent 100 μm. Asterisks indicate statistical significance (***P < 0.01 Mann-Whitney *U* test).

Parallel histological sections to those processed in Fig. 1 KA-induced astrocytic activation and inflammatory response in B6129-*Prnp*^0/0^ (n = 5), 129/Ola- *Prnp*^0/0^ (n = 5) mice at their respective controls (n = 5 each genotype) (Fig. 2a). Immunohistochemical analysis of GFAP-positive cells in the hippocampus of PrP^C^-null mice (either B6129 or 129/Ola) revealed large numbers of positive cells in the hippocampal CA1-3 when compared to *Prnp*^+/+^ controls (B6129-*Prnp*^0/0^ 75.7 ± 1.24 *vs* B6129-*Prnp*^+/+^ 43.4 ± 1.06; *P* = 0.005 Mann-Whitney *U* test. 129/Ola- *Prnp*^0/0^ 53.7 ± 1.44 *vs* 129/Ola-*Prnp*^+/+^ 42.9 ± 0.92; *P* = 0.0047 Mann-Whitney *U* test. Immunoreactive cells showed hypertrophic cell bodies and thicker glial processes (Fig. 2a). Moreover, quantitative real-time PCR data obtained from hippocampal samples 6 hours after KA-administration showed upregulation of the main pro-inflammatory markers TNFα (4.7-fold increase in B6129-*Prnp*^0/0^ (n = 3) *P* = 0.0046, Mann-Whitney *U* test and 3.8-fold increase in 129/Ola-*Prnp*^0/0^ (n = 3) (*P* = 0.0008, Mann-Whitney *U* test) *vs* respective wild type (B6129 n = 3 and 129/Ola n = 3) and IL1β (3.7-fold increase in B6.129- *Prnp*^0/0^ and 3.0-fold increase in 129/Ola- *Prnp*^0/0^ *vs* respective wild type (B6.129- *Prnp*^0/0^ *P* = 0.0046 and 129/Ola *Prnp*^0/0^ *P* = 0.0004, and Mann-Whitney *U* test)) (Fig. 2b).

### Chemical elimination of the GPI domain and of PrP^C^ reduces neuroprotection to KA in *Prnp*-transfected N2a cells

**Figure 3:**
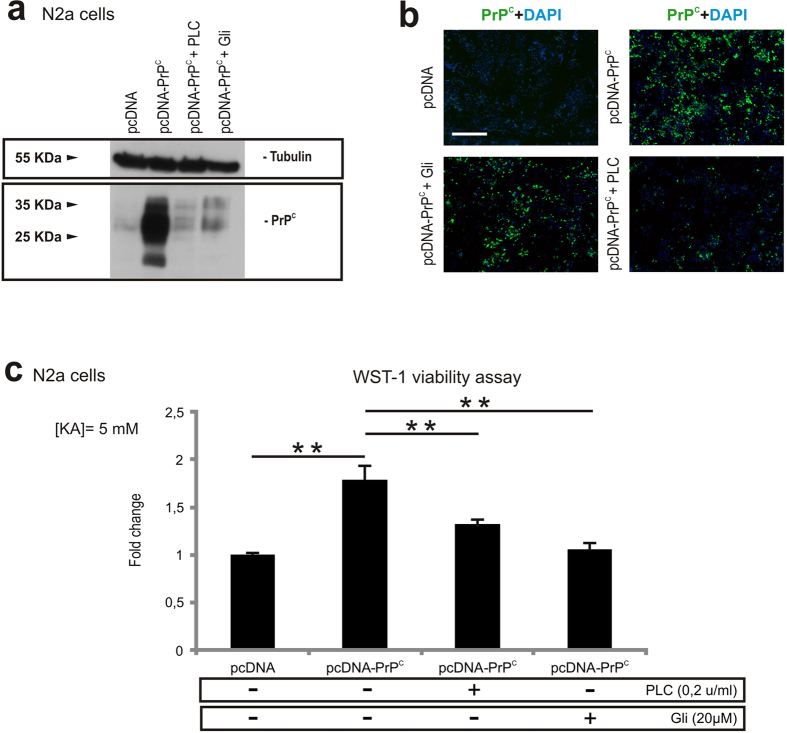
Chemical release of PrP^C^ from the cell surface impairs the neuroprotective function in KA-treated N2a cells. **(a**) Example of western blot determination of PrP^C^ levels in protein extracts of pcDNA, pcDNA-PrP^C^, pcDNA-PrP^C^ + PLC and pcDNA-PrP^C^ + Gli treated N2a cells. (**b**) Microphotographs illustrating PrP^C^ levels in cells transfected with pcDNA or pcDNA-PrP^C^, and treated with PLC (pcDNA-PrP^C^ + PLC) or Gli (pcDNA-PrP^C^ + Gli). PrP^C^ (green) was detected in non-permeabilized cells with 6H4 antibody. Note the decrease in PrP^C^ after PLC and Gli treatments in (**a**) and (**b**). (**c**) Histogram showing the decrease in surviving cells after KA-treatment (5 mM overnight) determined with the WST-1 viability test after PLC or Gli treatment of pcDNA-PrP^C^ transfected cells. Data (mean ± S.E.M.) were obtained from three independent experiments and represented as mean fold change. Scale bars represent 100 μm. Asterisks indicate statistical significance (**P < 0.05, Bonferroni *post hoc* test).

The GPI anchor in PrP^C^, as in other GPI-proteins, is not only necessary for the stability and attachment of the protein to the cell surface, but also for its association to specialized membrane microdomains (e.g., lipid rafts), its intracellular traffic and signal transduction events. In addition, the GPI group has been suggested as playing a role in prion disease toxicity, as transgenic mice expressing secreted forms of PrP^C^ lacking its GPI-moiety showed no clinical symptoms despite accumulating PrP^Sc^ in plaques^51^. Because in our microsatellite analysis the C57BL/10-PrP^GPIless^ mice (Tg44^+/+^ kindly provided by Dr. B. Chesebro) contained non-B10 regions in chromosome 3 and in chromosome 2 flanking the *Prnp* locus, we decided to check whether the degradation of the GPI binding domain of the PrP^C^ leading to a decreased PrP^C^ in the plasma membrane could overcome the neuroprotective function of PrP^C^ (Fig. 3a–c). In addition, this approach would determine whether the neuroprotective effect against KA treatment of PrP^C^ takes place at the membrane or intracellularly. Thus, N2a cells were treated either with Phospholipase C (PLC) enzyme or Glimepiride, a sulphonylurea approved for the treatment of diabetes mellitus, inducing the release of PrP^C^ from the surface of prion-infected neuronal cells, which releases PrP^C^ from the surface of neuronal cells^52^ (see methods for details). Decreased levels of PrP^C^ after treatments were detected with western blot (pcDNA = 0.39 ± 0.021; pcDNA-PrP^C^ = 0.91 ± 0.025; pcDNA-PrP^C^ + PLC = 0.45 ± 0.013; pcDNA-PrP^C^ + Gli = 0.496 ± 0.014) (Fig. 3a and Supplementary Fig. 2) or with immunocytochemistry in non-permeabilized cells (Fig. 3b) (pcDNA CTCF = 1.63 ± 0.09; pcDNA-PrP^C^ CTCF = 6.44 ± 0.12. pcDNA-PrP^C^ + Gli CTCF = 2.82 ± 0.20; pcDNA-PrP^C^ + PLC CTCF = 2.063 ± 0.15. pcDNA *vs* pcDNA-PrP^C^ *t* = 7.40, mean diff. = −4.8, 95% CI of diff. = −6.88 to −2.72. pcDNA-PrP^C^ *vs* pcDNA-PrP^C^ + Gli *t* = 5.31, mean diff. = 3.6, 95% CI of diff. = 1.43 to 5.78. pcDNA-PrP^C^ *vs* pcDNA-PrP^C^ + PLC *t* = 6.02, mean diff. = 4.37, 95% CI of diff. = 2.04 to 6.70. WST-derived results indicate that both treatments increased the sensitivity to KA (pcDNA *vs* pcDNA-PrP^C^ *t* = 4.38, mean diff. = −0.27, 95% CI of diff. = −0.44 to −0.10. pcDNA-PrP^C^ *vs* pcDNA-PrP^C^ + PLC *t* = 2.789, mean diff. = −0.175, 95% CI of diff. = −0.34 to −0.005. pcDNA-PrP^C^ *vs* pcDNA-PrP^C^ + Gli *t* = 4.46, mean diff. = 0.28, 95% CI of diff. = 0.11 to 0.45 (Fig. 3c).

### Enhanced cell death in the CA3 hippocampal region ΔF35 and ΔC4 mice compared to B6129 mice

**Figure 4:**
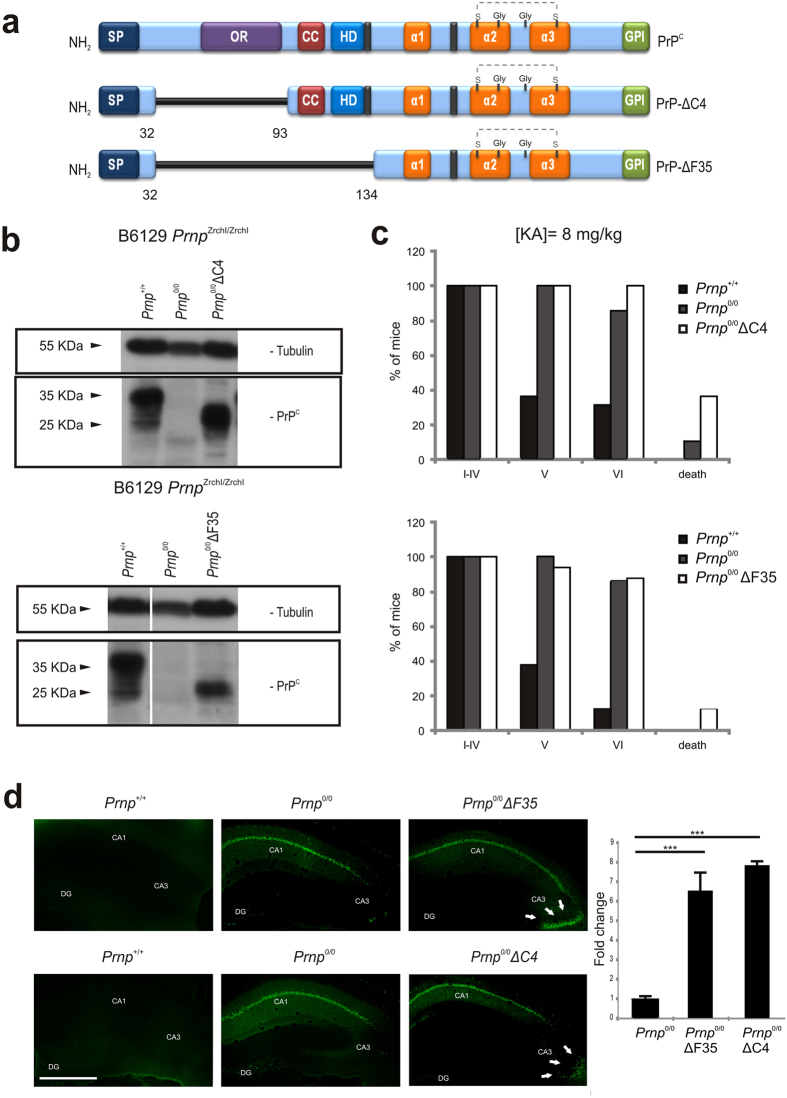
KA-induced seizures and neurotoxicity in ∆C4 and ∆F35 mice. (**a**) Scheme of PrP^C^ and N-terminal truncated forms overexpressed in B6129 *Prnp*^Zrchl/Zrchl^ *Prnp*^0/0^ ∆C4 and B6129 *Prnp*^Zrchl/Zrchl^ *Prnp*^0/0^ ∆F35 transgenic mice. (**b**) Example of western blot detection of PrP^C^ (6H4) expression in brain extracts obtained from untreated B6129 *Prnp*^Zrchl/Zrchl^ *Prnp*^+/+^and B6129 *Prnp*^Zrchl/Zrchl^ *Prnp*^0/0^, B6129 *Prnp*^Zrchl/Zrchl^ *Prnp*^0/0^ ∆C4 and B6129 *Prnp*^Zrchl/Zrchl^ *Prnp*^0/0^ ∆F35 mice. Tubulin was used as a loading control. (**c**) KA-induced seizure sensitivity in adult B6129 *Prnp*^Zrchl/Zrchl^ *Prnp*^0/0^ ∆C4 mice in comparison to B6129 *Prnp*^Zrchl/Zrchl^ *Prnp*^+/+^ and B6129 *Prnp*^Zrchl/Zrchl^ *Prnp*^0/0^ (upper panel). KA-induced seizure sensitivity in 5–7 week-old B6129 *Prnp*^Zrchl/Zrchl^ *Prnp*^0/0^ ∆F35 mice in comparison to B6129 *Prnp*^Zrchl/Zrchl^ *Prnp*^+/+^, and B6129 *Prnp*^Zrchl/Zrchl^ *Prnp*^0/0^ (lower panel). Bars represent the percentage of mice reaching each stage. (**d**) Fluoro-Jade B staining in hippocampal sections of B6129 *Prnp*^Zrchl/Zrchl^ *Prnp*^+/+^, B6129 *Prnp*^Zrchl/Zrchl^ *Prnp*^0/0^, B6129 *Prnp*^Zrchl/Zrchl^ *Prnp*^0/0^ ∆C4 and B6129 *Prnp*^Zrchl/Zrchl^ *Prnp*^0/0^ ∆F35 mice 24 hours after KA treatment (8 mg/kg b.w.). The quantification of the Fluoro-Jade B-positive cells in the CA3 region is shown in the right plot. Scale bars represent 200 μm. Abbreviations: DG, Dentate gyrus; CA1-3, hippocampal regions 1 and 3; h, hilus; gl, granule cell layer; ml, molecular layer; sr, *stratum radiatum*; slm, *stratum lacunosum-moleculare*; sl, *stratum lucidum*; sp, *stratum pyramidale*; so, *stratum oriens*. Asterisks indicate statistical significance (***P < 0.01, Bonferroni *post hoc* test).

*Prnp*^0/0^ ΔF35 (ΔF35) and *Prnp*^0/0^ ΔC4 (ΔC4) mice (Fig. 4a) were generated some years ago by nuclear injections of constructs into fertilized oocytes from B6129 *Prnp*^Zrchl/Zrchl^ mice^53^. The number of copies of the construct was estimated as 25 for ΔC4 and 70 for ΔF35 mice. However, brain extracts obtained from these mice revealed similar levels of PrP−ΔC4 (*Prnp*^*+/+*^ = 0.755 ± 0.075; PrP−ΔC4 = 0.81 ± 0.03; *P* = 0.4; CI = 95%, Mann-Whitney *U* test) to wild-type but lesser amounts of PrP−ΔF35 than wild-type (*Prnp*^*+/+*^ = 0.91 ± 0.01; PrP−ΔF35 = 0,70 ± 0.021; *P* = 0.0048, CI = 95%, Mann-Whitney *U* test) (Fig. 4b and Supplementary Fig. 3). Nevertheless only ΔF35 mice showed cerebellar degeneration at around 60 days of life^53^ (Supplementary Fig. 4). Irrespective of the cell type, the expression of the truncated form may induce *per se* cell death *in vivo*^54^ as well as *in vitro*^55^. Thus in the next experiments we treated these mice with 8 mg/kg b.w. of KA following the above-mentioned protocol. Behavioural results reported a similar evolution between ΔC4 (n = 11) and ΔF35 (n = 15) mice compared to B6129 *Prnp*^Zrchl/Zrchl^ *Prnp*^0/0^ (n = 35) and B6129 *Prnp*^*+/+*^ (n = 30) (Fig. 4c and Supplementary Movie 4). However, the number of dead was greater in ΔC4 (≈38%) and ΔF35 (≈15%) compared to B6129 *Prnp*^Zrchl/Zrchl^ *Prnp*^0/0^ mice. These results also correlate with an increased presence of Fluoro-Jade B cells observed in histological sections of the hippocampus, especially in the CA3 region (ΔF35 = 87.12 ± 0.41; ΔC4 = 104.62 ± 0.088; B6129 *Prnp*^Zrchl/Zrchl^ = 13,37 ± 0.055. B6129 *Prnp*^Zrchl/Zrchl^ *vs* ΔF35 *t* = 7.577; mean diff. = −73.75, 99% Cl of diff. = −115.4 to −32.1. B6129 *Prnp*^Zrchl/Zrchl^ *vs* ΔC4 *t* = 9.375; mean diff. = −91.25, 99% CI of diff. = −132.9 to −49.6, Bonferroni *post hoc* test). (Fig. 4d). Taken together, these results indicate that the expression of the truncated form including the octarepeat region (OR) with or without the Central Domain (CD) of PrP^C^ potentiates the effects of the KA (<10 mg/kg b.w.), and they highlight the octarepeat domain as a key candidate in the neuroprotective functions of PrP^C^.

### Acute transfection of pcDNA-PrP^∆CD^ but not pcDNA-PrP^∆F35^ protects N2a cells from KA excitotoxicity *in vitro*

**Figure 5:**
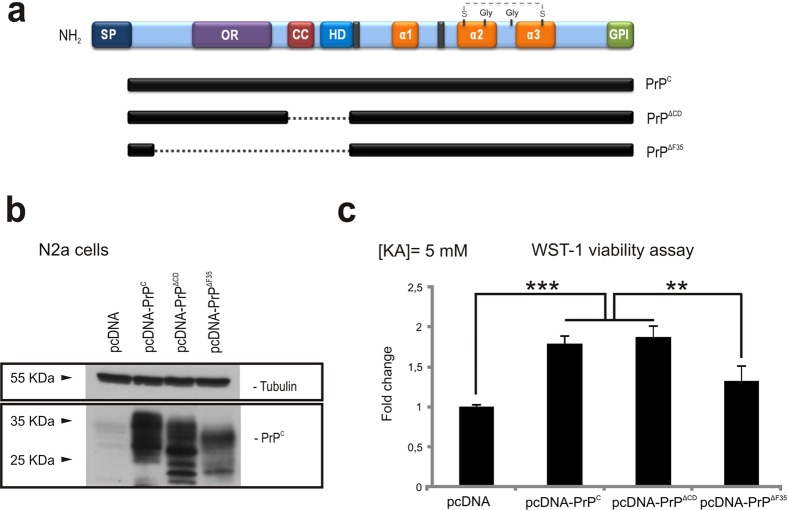
KA-excitotoxicity in N2a cells transiently transfected with PrP^C^ or PrP-∆CD and PrP-∆F35 constructs. (**a**) Scheme illustrating domain organization in PrP^C^ and its related PrP^∆CD^ and PrP^∆F35^ mutants. (**b**) Example of western blot determination of the different PrP^C^ constructs employed in the experiment. (**c**) WST-1 viability assay performed over N2a cells previously transfected with pcDNA3.1 (empty vector, pcDNA), pcDNA-PrP^C^, pcDNA-PrP^∆CD^ and pcDNA-PrP^∆F35^ and treated overnight with 5 mM KA. Data were normalized with untreated controls. A clear reduction in cell viability was observed in pcDNA-PrP^∆F35^ transfected cells when compared to those overexpressing pcDNA-PrP^C^. In contrast, pcDNA-PrP^∆CD^ transfected cells showed similar viability to pcDNA-PrP^C^ transfected cells. Histograms represent the mean ± S.E.M. of three different experiments. Asterisks indicate statistical significance (**P < 0.05, ***P < 0.01, Bonferroni *post hoc* test).

In order to corroborate *in vitro* the participation of the OR in PrP^C^-mediated neuroprotection to KA, we performed a viability assay using N2a cells. Cells were transfected with vectors encoding either the full length of *Prnp* or two truncated forms lacking CD (PrP^∆CD^, residues 95–133), which bridge the flexible amino proximal tail and the globular carboxy proximal domain, or else a longer deletion including the central domain (CD) plus the OR (PrP^∆F35^, residues 32–134) (Fig. 5a). After transfection, levels of PrP^C^ and its truncated forms in transfected cells were checked with western blotting (Fig. 5b and Supplementary Fig. 5). In addition, the expression of the truncated forms was strictly modulated to avoid inducing cell death during the experiments. Transfected cells were treated overnight with 5 mM KA and further processed to WST-1 assays. Colorimetric WST-1 assay showed that PrP^C^ and PrP^∆CD^ transfection increased cell culture viability after KA treatment, an effect that was not observed in PrP^∆F35^ transfected cells (pcDNA = 0.54 ± 0.005; pcDNA-PrP^C^ = 0.94 ± 0.06; pcDNA-PrP^**∆CD**^ = 1.047 ± 0.012 and pcDNA-PrP^∆F35^ = 0.88 ± 0.03. pcDNA *vs* pcDNA-PrP^C^ *t* = 36.18; mean diff. = −0.4, 99% CI of diff. = −0.45 to 0.34. pcDNA *vs* pcDNA-PrP^**∆CD**^ *t* = 45.83; mean diff. = −0.506, 99% CI of diff. = −0.55 to 0.45. pcDNA-PrP^**∆CD**^ *vs* pcDNA-PrP^**∆**F35^ *t* = 15.08, mean diff. = 0.166, 95% CI of diff. = 0.1282 to 0.20). pcDNA-PrP^C^ *vs* pcDNA-PrP^**∆**F35^ *t* = 5.42, mean diff. = 0.06, 95% CI of diff. = 0.021 to 0.09) (Fig. 5c). These results reinforce the idea of the OR domain participating in the described neuroprotective role of PrP^C^ to excitotoxic damage in N2a cells.

## Discussion

### Role of PrP^C^ in neuroprotection against KA

The diversity of phenotypic changes described in *Prnp*-knockout mice has hindered the study of the physiological function of PrP^C10^. In fact the reported differences between *Prnp*^+/+^ and *Prnp*^0/0^ mice have led to certain controversial results, especially in terms of electrophysiology and susceptibility to excytotoxic insults. Several pieces of evidence, including some from previous studies by our group using B6.129 *Prnp*^Zrchl/Zrchl^ 2,18,21,40, support the idea that *Prnp*-knockout mice are more susceptible to KA, NMDA and PTZ, exhibiting an enhanced epileptic response and neurotoxicity in the hippocampus when compared to wild type controls^17^,^19^. Contradictory results have been published by other groups, who described an elevated threshold for epileptiform activity in *Prnp*^0/0^ hippocampal slices exposed to bicuculline, PTZ or zero-magnesium conditions^24^. Similar discrepancies have been found when analysing neurotransmission-associated parameters in mice devoid of PrP^C34,35,37,38^. Due to this lack of reproducibility between groups, the possibility that reported phenotypes could be attributed to external factors, such as different mice strains (e.g.,^25^ or 32) or experimental procedures (age of mice, KA concentration, treatments, etc.) must be strongly considered. Indeed, age-dependent loss of LTP in PrP^C^-null mice^56^,^57^ has also been identified.

More recently, a comparative analysis between *Prnp*-knockout mouse strains has led to the proposal that the increased sensitivity to KA-induces seizures in *Prnp*-knockout mice is not associated with the absence of the protein but rather with inappropriate comparison with wild-type controls that differ in genetic background^22^,^23^,^58^. In this study, background influence in susceptibility to excitotoxic damage was corroborated by evaluating the onset of epileptic seizure of wild-type mice from B6129, B6.129,129/Ola and FVB/N strains. Our results show that FVB/N wild-type mice showed more seizures than B6129 and 129/Ola mice, corroborating previous studies^25^,^26^,^59^. In addition, 129/Ola wild-type showed increased expression of PrP^C^ that may also reduce the number of grade VI seizures.

In our study we observed the relevant effects of intraperitoneal injection of KA in B6129 *Prnp*^Zrchl/Zrchl^ *Prnp*^0/0^ (with ≈46% 129 microsatellites), B6.129 *Prnp*^Zrchl/Zrchl^ *Prnp*^0/0^ (with ≈6–7% 129 microsatellites) and 129/Ola-*Prnp*^0/0^ mice (with 100% 129 background) compared with the appropriate wild-type controls. Results indicate a decline in the epileptic seizures when low numbers of 129 microsatellites are present in B6.129 *Prnp*^Zrchl/Zrchl^ *Prnp*^0/0^. These results point to polymorphisms of some of the “*Prnp-* flanking genes” as masking the participation of PrP^C^ in neuroprotection, as reported in other processes^30^. Analysis of the 9 genes functions using gene ontology and Pubmed searches indicates that Prex1^60^, SIRPα^61^, Traf1^62^, Thbs1^63^,^64^, Rmdn3^65^, Tyro3^66^,^67^, Slc30a4^68^, Mertk^69^ and B2m^70^ are involved in neurotransmission, LTP and neuroprotection. Whether polymorphisms in these genes participate in these effects warrants further study. However, the phenotypic differences observed between 129/Ola *Prnp*^+/+^ and 129/Ola *Prnp*^0/0^ mice could only be associated with *Prnp*-deletion, as no possible genetic variability exists between the two. These results differ from those recently published by Striebel and coworkers^23^ who didn’t observe PrP^C^-linked neuroprotection despite using the same mouse strain. These contradictory results may be due to KA-dosage or to minor differences in the experimental procedure. Furthermore, we must not forget that this neuroprotective property of PrP^C^ is further supported by other *in vivo* models, such as transient knockdown of *Prnp* homologs in zebrafish^71^, and also some *in vitro* experimental approaches including PrP^C^ downregulation and detachment of the plasma membrane in neuronal primary cultures and neuroblastoma cell lines (18,19,20 and present results).

In conclusion, our findings support the notion that PrP^C^ is involved in neuroprotection to KA-induced seizures and excitotoxicity and that it actively participates in the increased epileptic response observed in mice devoid of PrP^C^. In parallel, as yet unknown factors associated with *Prnp*-flanking genes also affect KA susceptibility.

### The neuroprotective function of PrP^C^ depends on membrane anchoring

Our results indicate that the neuroprotective function of PrP^C^ against KA in N2a cells depends on membrane anchoring. The interaction of different regions of PrP^C^ with plasma membrane through the GPI domain has been considered necessary to induce the clinical symptoms in GPI-negative transgenic mice (C57BL/10-PrP^GPIless^ mice)^51^,^72^. In fact, the injection of antibodies directed to the α1 and α3 regions of the PrP^C^ induces neurotoxic effects^73^. These results have also been corroborated in newly developed mice lacking the globular domain (FTgpi mice)^74^. In fact, these results corroborate previous observations^75^. Taken together, these studies suggest that the proximity of the flexible tail (N-terminal domain) to the plasma membrane triggers intracellular oxidative stress responses leading to cell death^74^,^76^. Under this scenario we can consider that our data reinforce the idea that the integrity of the N-terminal domain is mandatory for neuroprotection (see below) as well as the notion that membrane interaction is a necessary part of the neuroprotective function reported *in vivo*.

### The neuroprotective function of PrP^C^ is abolished in the absence of the OR of PrP^C^

Structural analysis of PrP^C^ architecture has determined different functionally relevant domains in this protein (see^77^,^78^ for review). Besides the highly conserved hydrophobic domain, the flexible and unstructured N-terminus region includes a copper binding site consisting of four tandem repeats of the sequence PHGGGWGQ, which seems to be involved in the endocytic process of the protein^79^ and copper homeostasis^3^. In fact a recent study indicates a relevant role of copper binding in the neuroprotective function of PrP^C^ modulating NMDA receptor^48^. In our study we developed *in vivo* as well as *in vitro* tests to check the involvement of the OR domain as a mediator of PrP^C^ neuroprotective function in a model of KA treatment. The behavioural and histological data presented here by ΔC4 and ΔF35 mice revealed increased cell death in the hippocampus of the KA injected mice compared to B6129 *Prnp*^0/0^ (genetic background of these mice). In parallel, the CD is not involved in the neuroprotective functions of PrP^C^ (at least in N2a cells) since their absence does not modify these properties when compared to full length PrP^C^, in contrast to the absence of the OR regions.

The overexpression of PrP−ΔF35 in a B6129 *Prnp*^Zrchl/Zrchl^ *Prnp*^0/0^ background leads to cerebellar neurodegeneration^53^, but not hippocampal degeneration^55^ (Supplementary Fig. 4). In contrast, ΔC4 mice (with similar background) do not show cerebellar or hippocampal degeneration, but when subjected to controlled ischemia show significantly greater oxidative stress damage when compared to wild type mice^80^. This also happens when PrP−ΔF35 is overexpressed in HEK293 cells, leading to increased Caspase 3 activity in transfected cells and cell death^55^. Truncated forms of PrP^C^ lacking the OR interfere with PrP^C^ endocytosis via clathrin-coated vesicles and beta-cleavage of PrP^C^, respectively, thereby impairing the antioxidative functions of PrP^C^^81^,^82^. Thus it is reasonable to consider that cells with an intrinsic deficit in oxidative stress homeostasis may also be more prone to KA treatment if the appropriate KA receptors are also expressed, as happens in the hippocampus^83^,^84^,^85^.

As indicated above, using antibody-mediated degeneration, Sonati *et al.,* demonstrated that ligands directed to the α1 and α3 helices of the PrP^C^ globular domain induce cerebellar cell death by activating oxidative stress that can be overcome by deletions in the OR region^73^. This also happens in FTgpi mice lacking the α1–α3 helix region of the PrP^C^ 74. In addition, mice lacking the ΔCD also reported white matter pathology and peripheral neuropathy^86^. Surprisingly, the reported degeneration of the ΔCD mice could be reversed by coexpression of PrP^C^ lacking all octarepeats^86^. These data are in contrast to a recent study indicating that the antibody ICSM18 (recognizing aa143–153 of PrP^C^) does not induce cell death^87^. In our experiments, we observed that cells transfected with PrP−ΔCD are able to overcome KA-mediated cell death as are those transfected with full length PrP^C^, in contrast to PrP−ΔF35 transfected N2a cells. Our *in vitro* experiments are different from the *in vivo* situation, since an effect of ligands in the CD regions is unlikely; rather, they suggest a parallel effect of KA excytotoxicity plus the homeostatic imbalance induced by the absence of the 32–93 region of the overexpressed PrP^C^. Despite the existing data, the precise mechanism underlying OR-dependent neuroprotection remains to be elucidated in the above-mentioned studies^73^,^87^,^88^.

In conclusion, our study dissects the effects of the intraperitoneal injection of various doses of KA in several *Prnp* mouse models and indicates that: i) PrP^C^ plays a role in neuroprotection in KA-treated cells and mice; ii) for this role PrP^C^ should be linked to the membrane; iii) polymorphisms of some unidentified “*Prnp*-flanking genes” play a parallel role to PrP^C^ in the KA-mediated responses in B6129 and B6.129 *Prnp*^Zrchl/Zrchl^ *Prnp*^0/0^ mice; and iv) the absence of the aa32–93 region negatively affects the neuroprotective function of PrP^C^ in KA-treated cells.

## Methods

### Reagents and antibodies

KA, glimepiride and phospholipase C were purchased from Sigma (Poole Dorset, UK). Fluoro-Jade B was from Millipore (Billerica, MA), SYBR green (Applied Biosystems, USA) and WST−1 reagents were from Roche (Basel, Switzerland). Lipofectamine plus was from Invitrogen (Carlsbad, CA). The following antibodies were used in this study: Anti-PrP SAF61 mouse monoclonal antibody (1:1000 diluted) antibody was from Spi-Bio (Cayman Chemical, Massy, France) and anti-PrP 6H4 mouse monoclonal antibody (1:5000 diluted western blotting and 1:250 immunocytochemistry) antibody was from Prionics (Schlieren, Switzerland). Mouse monoclonal antibody anti-tubulin (1:10000 diluted) was from Sigma (Poole Dorset, UK) and rabbit-raised polyclonal antibody against GFAP (1:500 diluted) was from Millipore (Billerica, MA).

### Animals

Adult male C57Bl6/129sv-*Prnp*^0/0^ (B6129 *Prnp*^Zrchl/Zrchl^ Zurich I) mice were purchased from the European Mouse Mutant Archive (EMMA, Monterotondo, Italy). The FVB/N animals were either wild-type or homozygous for the deletion of *Prnp* gene. 129/Ola *Prnp*^Edbg/Edbg^ *Prnp*^+/+^ and 129/Ola *Prnp*^0/0 9^ were obtained from Dr. J. Manson (Edinburgh). B6129 *Prnp*^Zrchl/Zrchl^ mice were backcrossed for 8 generations to obtain 6–7% of 129 microsatellites (B6.129 *Prnp*^Zrchl/Zrchl^). Transgenic B6129 *Prnp*^0/0^ PrP^∆C4^ 89 and B6129 *Prnp*^0/0^ PrP^∆F35^ 53 were obtained from Prof. A. Aguzzi. To avoid putative background-specific differences between mice, all of the experiments were conducted using littermates derived from heterozygous (*Prnp*^+/0^ ΔF35^+^) and *Prnp*^0/0^ parents. Specific primers for *Prnp* genotyping were designed in our laboratory based on the original P10 and P3 primers described elsewhere^8^. PrP^∆F35^ transgene was detected by using 5′-CCTGGGACTCCTTCTGGTACCGGGTGACGC-3′ and 5′-CAACCGAGCTGAAGCATTCTGCCT-3′ set of primers and PrP^∆C4^ with 5′-GGCTGGGCTTGTTCCACTGATTATGGG-3′ and 5′-CAACCGAGCTGAAGCATTCTGCCT-3′. Tg44^+/+^ mice expressing anchorless PrP^C 51^ on a C57Bl/10 background were obtained from Dr. Bruce Chesebro (Laboratory of Persistent Viral Diseases, Rocky Mountain Laboratories, National Institute of Allergy and Infectious Diseases, Hamilton, MT 59840, USA). A total of 83 litters (193 animals) were used in the present study. All experiments were performed under the guidelines and protocols of the Ethical Committee for Animal Experimentation (CEEA) of the University of Barcelona, and the protocol for the use of animals in this study was reviewed and approved by the CEEA of the University of Barcelona (CEEA approval #115/11 and 141/15).

### KA administration in mice and seizure analysis

Convulsive non-lethal seizures in mice were induced by administration of KA in a multiple dose protocol. Fresh KA solution was prepared for each experiment. Animals were weighed and intraperitoneally injected with 8 or 10 mg/kg KA (b.w.) dissolved in 0.1 M PBS, pH 7.2. at 0 min, 30 min and 60 min. In parallel mice, 0.1 M PBS, pH 7.2 was injected as control (vehicle). Adult (2–3 months old) animals were used in all experiments except for B6129 *Prnp*^Zrchl/Zrchl^ *Prnp*^0/0^ PrP^∆C4^ PrP^∆F35^ and their respective controls, which were used at 5–7 weeks-old due to their described early lethality^53^.

After KA-injection mice were distributed in boxes (1–5 mouse/box) and the behaviour of the mice was recorded for 4 hours using a digital video camera (SONY DCR-HC30E Digital video camera). Seizure intensity was evaluated during the 4 hours after the first injection using the following criteria: grades I-II: hypoactivity and immobility, grades III-IV: hyperactivity and scratching, grade V: loss of balance control and intermittent whole-body convulsions and grade VI: continuous seizures and bouncing activity (commonly referred to as ‘popcorn’ behaviour). The characterization of seizure intensity was developed in two stages. First seizures were analysed *in situ* after KA-injections in the Molecular and Cellular Neurobiotechnology Laboratory (IBEC) by P.C., F.Ll. and A.M-A. (B6129 *Prnp*^Zrchl/Zrchl^ Zurich I, B6.129 *Prnp*^Zrchl/Zrchl^, B6129 *Prnp*^0/0^ PrP^∆C4^, B6129 *Prnp*^0/0^ PrP^∆F35^, FVB/N *Prnp*^0/0^ mice strains and its respective controls) and in the Centro de Investigación en Sanidad Animal (CISA-INIA) by P.C, P.A-C., J.C.E. (129/Ola *Prnp*^0/0^ and 129/Ola *Prnp*^Edbg/Edbg^ *Prnp*^+/+)^. After this initial characterization that included time of the appearance of seizures, scale of seizures, etc, video recordings of each experiment were analysed by J.A.D.R. at IBEC and J.M.T. at CISA-INIA without knowledge of the results of the previous *in situ* analysis. Afterwards, a contingency table was generated by comparing both analyses and the final data were plotted. Grades I to IV were grouped together for better data representation, as all animals tested reached these four epileptic stages. The statistical analysis of the obtained data was performed using Mann–Whitney *U* non-parametric test using Prism 5.0c (Mac OsX, Grahpad). Data are presented as percentage or as mean ± standard error of the mean (S.E.M.). A value of ***P < 0.01 was considered statistically significant.

### Western blotting

Brain samples from non-treated mice were homogenized (10% wt/vol) in ice-cold lysis buffer—50 mM Tris-HCl, pH 7.4, 150 mM NaCl, 0.5% (wt/vol) Triton X-100, 0.5% (wt/vol) Nonidet P-40 (IGEPAL; Sigma), glycerol 10%, 1 mM EDTA, 1 mM EGTA, and protease and phosphatase inhibitors—using a motor-driven, glass-Teflon homogenizer in ice, and then centrifuged at 15,000*g* for 20 min. Protein concentration was quantified with BCA kit (Pierce). Protein extracts were boiled in Laemmli sample buffer at 100 °C for 5 min. Equal amounts of total protein were separated with 6–10% SDS-PAGE electrophoresis, electrotransferred to polyvinylidene fluoride (PVDF) membranes (Millipore), and probed with indicated antibodies. Visualization of bound antibodies was performed using goat anti-mouse HRP (1:4000 diluted; Dako, Glostrup, Denmark) and the ECL Plus kit (Amersham-Pharmacia Biotech, GE Healthcare Bio-Sciences, Piscataway, NJ, USA).

### Densitometry and statistical processing of processed films

For quantification, developed films were scanned at 2400 × 2400 dpi (i800 MICROTEK high quality film scanner), and the densitometric analysis of the different PrP^C^ bands was performed in each case using Quantity One Image Software Analysis (Biorad). Each densitometric value of PrP^C^ and truncated form ΔC4, ΔF35 and ΔCD (0–255 gray scales) was normalized with the corresponding Tubulin densitometric values (0–255 gray scale). Three different experiments were used in each analysis unless specified. The statistical analysis of the obtained data was performed using Bonferroni *post hoc* test (Multiple comparison test) or Mann-Whitney *U* non-parametric test using Prism 5.0c (Mac OsX, Grahpad). Data are presented as mean ± standard error of the mean (S.E.M.). A value of **P < 0.05 was considered statistically significant.

### Fluoro-Jade B staining

Mice were perfused with 4% paraformaldehyde dissolved in 0.1 phosphate buffer, pH 7.3 24 hours after the first KA injection, post-fixed overnight in the same fixative, and cryoprotected in 30% sucrose. 30 μm-thick coronal brain sections were obtained in a freezing microtome (Leica, Wetzlar, Germany). Sections containing dorsal hippocampus (Bregma = −1.2 to −1.9^90^) were rinsed for 2 h in 0.1 M Tris, pH 7.4, mounted and air dried at room temperature overnight. The next day, sections were pre-treated for 3 min in absolute ethanol, followed by 1 min in 70% ethanol and 1 min in distilled water. They were then oxidized in a solution of 0.06% KMnO4 for 15 min. After three rinses of 1 min each in distilled water, the sections were incubated for 30 min in a solution of 0.001% Fluoro-Jade B (Chemicon) containing 0.01% of DAPI (Sigma) in 0.1% acetic acid. The slides were rinsed in deionized water for 3 min each, dried overnight, cleared in xylene, cover-slipped with Eukitt (Merck, Darmstadt, Germany) and examined using an Olympus (Hamburg, Germany) BX61 epifluorescence microscope. The statistical analysis of the obtained data was performed using Bonferroni *post hoc* test (Multiple comparison test) using Prism 5.0c (Mac OsX, Grahpad). Data are presented as mean ± standard error of the mean (S.E.M.). A value of ***P < 0.01 was considered statistically significant.

### Histology and immunofluorescence

For histology, mice were perfused with phosphate buffered 4% paraformaldehyde, pH 7.3 24 hours after the first KA injection, post-fixed overnight in the same fixative, and cryoprotected in 30% sucrose as above. A freezing microtome (Leica, Wetzlar, Germany) was used to obtain 30 μm-thick coronal sections, which were rinsed in 0.1 M PBS before 1 hour’s incubation at room temperature in 0.1 M PBS containing 0.2% gelatin, 10% normal goat serum, 0.2% glycine, and 0.2% Triton X-100. Sections were then incubated overnight at 4 °C with indicated primary antibodies. After washing in 0.1 M PBS containing 0.2% Triton X-100, sections were incubated with goat anti-rabbit Alexa Fluor 568-tagged secondary antibody (1:200 diluted; Molecular Probes, Eugene, OR, USA), washed in 0.1 M, PBS and mounted in Fluoromount (Vector Labs, Burlingame, CA, USA). Immunohistochemical controls, which included omission or substitution of primary anti-GFAP antibody by either normal rabbit serum prevented immunostaining. For quantification of GFAP-positive astrocytes in the stratum radiatum of the dorsal hippocampal region, immunoreacted sections (5 sections of each mouse, n = 5 mice per genotype) were photodocumented with an Olympus BX61 fluorescence microscope equipped with a cooled DP12L camera. Photomicrographs were obtained using a 40X objective with identical time exposure (100–150 ms) between preparations from each wild-type and respective knockout mouse. No modifications were applied to the obtained pictures. Numbers of GFAP-expressing cells were determined by counting positive cells in five frames (250 × 200 μm) corresponding to the hippocampal CA1-3 regions of five mice of each genotype. Data were expressed as mean ± standard error of the mean (S.E.M). The statistical analysis of the obtained data was performed using Mann–Whitney *U* non-parametric test using Prism 5.0c (Mac OsX, Grahpad). A value of *P* < 0.01 was considered statistically significant.

### RT-qPCR

Total RNA from hippocampal samples obtained from treated (6 h after KA-administration) and non-treated mice was purified with the mirVana isolation kit (Ambion, Austin, TX, USA) and used to make the single-stranded cDNAs required as templates for the RT-qPCR amplification. Sets of primers used in this study were: for TNFα 5′- AGCAAACCACCAAGTGGAGGA- 3′ and 5′-GCTGGCACCACTAGTTGGTTGT- 3′; and for ILβ 5′- TTGTGGCTGTGGAGAAGCTGT- 3′ and 5′- AACGTCACACACCAGCAGGTT- 3′. The reaction was performed with the Roche LightCycler 480 detector, using 2x SYBR Green Master Mix (Roche) as reagent, as indicated by the manufacturer. Amplification protocol consisted of a denaturation-activation cycle (95 °C for 10 min) followed by 40 cycles of denaturation-annealing-extension (95 °C, 15 sec; 60 °C, 40 sections; 72 °C, 5 sec; 98 °C, continuous). LightCycler 480 software was used for mRNA quantification. The data were analysed using the *ΔΔ*Ct method, which provides the target gene expression values as fold changes in the problem sample compared with a calibrator sample. Both problem and calibrator samples were normalized by the relative expression of a housekeeping gene (glyceraldehyde-3-phosphate dehydrogenase [GAPDH]). These analyses developed 3 different samples. The statistical analysis of the obtained data was performed using Mann–Whitney *U* non-parametric test using Prism 5.0c (Mac OsX, Grahpad). Data are presented as mean ± standard error of the mean (S.E.M.). A value of ***P < 0.01 was considered statistically significant.

### Cell culture and treatments

The murine neuroblastoma cell line Neuro2a (N2a) expressing low levels of PrP^C^ was grown at 37 °C, 5.5% CO2 in Dulbecco’s modified Eagle’s medium (DMEM) supplemented with 4.5 g/L glucose, 10% fetal bovine serum (FBS) and antibiotics (Invitrogen-Life Technologies, Barcelona, Spain). For vector transfection, cells were plated at 1 × 10^5^ cells/well in a 24-well plate and transiently transfected the next day using Lipofectamine 2000 reagent in Optimem medium, as indicated by the manufacturer (Invitrogen-Life Technologies). Four hours after transfection, cells were washed and the medium was replaced with DMEM containing 10% FBS. PrP^C^ expression after transfection was checked with western blot analysis. In a first set of experiments, N2a cell cultures were transiently transfected with pcDNA and pcDNA-PrP^C^ and maintained *in vitro* at 37 °C, 5.5% CO_2_. Cells were deprived for 24 h and treated with Glimepiride 20 μM and 0.2 u/ml PLC (final concentration). We used both treatments since some reports indicate that Glimepiride may act on ATP-dependent K+ channels that may also affect cell survival^91^. One hour later, 5 mM KA dissolved in 0.1 M PBS was added to the media. After treatment, cultures were rinsed twice in KA-free culture medium, and cell viability was determined with WST-1 viability assay (see below). In parallel, non-permeabilized cells were processed to PrP^C^ detection by immunofluorescence using the 6H4 antibody and a goat anti-mouse Alexa Fluor 488–tagged secondary antibody (1:200 diluted; Molecular Probes). For fluorescence quantification of cell-bound Alexa Fluor 488, immunoreacted cultures (n = 10 per experimental group) were photodocumented with an Olympus BX61 + DP12L camera. Photomicrographs were obtained using a 20X objective with identical time exposure (250–300 ms) for preparations from each group. No modifications were applied to the obtained pictures. Fluorescence intensity was determined using ImageJ by measuring the corrected total cell fluorescence (CTCF) as: CTCF = integrated density – (area of selected N2a mesured cells x mean fluorescence of background). Data were expressed as mean ± standard error of the mean (S.E.M). The statistical analysis of the obtained data in these experiments was performed using Bonferroni *post hoc* test (Multiple comparison test) using Prism 5.0c (Mac OsX, Grahpad). A value of *P* < 0.05 was considered statistically significant. These experiments were repeated four times. In a second set of experiments, N2a cells were transfected with pcDNA3.1, pcDNA3.1-PrP^C^, pcDNA3.1-PrP^∆CD^ and pcDNA3.1-PrP^∆F35^. KA (5 mM) treatment was carried out on serum-deprived cells 24 h after transfection. Cell viability was determined using a commercially available WST-1-based assay. Cell cultures were incubated with WST-1 reagent for 2 hours. Then absorbance at 450 nm was measured in a multiwell plate reader (Merck ELISA System MIOS). Data were normalized with *A*450 in untreated controls. pcDNA3.1-PrP^C^, pcDNA3.1-PrP^∆CD^ and pcDNA3.1-PrP^∆F35^ were kind gifts from Prof. D. Harris (Boston University) and Prof. A. Aguzzi (University Hospital of Zurich). These experiments were repeated five times. The statistical analysis of the obtained data in these experiments was performed using Bonferroni *post hoc* test (Multiple comparison test) using Prism 5.0c (Mac OsX, Grahpad). Data are presented as mean ± standard error of the mean (S.E.M.). Values of **P < 0.05 and ***P < 0.01 were considered statistically significant.

## Additional Information

**How to cite this article**: Carulla, P. *et al.* Involvement of PrP^C^ in kainate-induced excitotoxicity in several mouse strains. *Sci. Rep.* **5**, 11971; doi: 10.1038/srep11971 (2015).

## Supplementary Material

Supplementary movie 1

Supplementary movie 2

Supplementary movie 3

Supplementary movie 4

Supplementary Information
