# Supplementary material for: Involvement of PrPC in kainate-induced excitotoxicity in several mouse strains
Source: Sci Rep. 2015 Jul 9;5:11971. doi: 10.1038/srep11971 (PMC4648388; doi:10.1038/srep11971)
Supplement: Supplementary Information [file srep11971-s5.pdf]

SUPPLEMENTARY INFORMATION FOR EDITORIAL PURPOSES

REF: SREP-15-02633-B

Title: Involvement of PrPC in kainate-induced excitotoxicity in several mouse strains

Authors: Patricia Carulla, Franc Llorens, Andreu Matamoros-Angles, Patricia Aguilar-Calvo, Juan Carlos Espinosa, Rosalina Gavín, Isidre Ferrer, Giuseppe Legname, Juan Maria Torres, José A. del Río

Supplementary Figures/Movies legends:

Supplementary Tables 1-6: Description of the seizure level reached by each mouse plotted in the Figure 1a.

Supplementary Figure 1: Full size Western blot corresponding to Figure 1b for Tubulin and PrP^{C} levels in brain extracts from B6129 $Prnp^{Zrchl/Zrchl}$ $Prnp^{+/+}$, B6129 $Prnp^{Zrchl/Zrchl}$ $Prnp^{0/0}$, 129/Ola $Prnp^{Edbg/Edbg}$ $Prnp^{+/+}$, 129/Ola $Prnp^{Edbg/Edbg}$ $Prnp^{0/0}$, FVB/N $Prnp^{+/+}$ and FVB/N $Prnp^{0/0}$ mice. (**a**) 1 minute exposure. (**b**) 5 minutes exposure. L points to the ladder lines. Notice that the L lines were omitted in Fig. 1b. Red arrows points the portions of the Western blot showed in the Figure 1b.

Supplementary Figure 2: Full size blot corresponding to Figure 3a for Tubulin and PrP^{C} levels in protein extracts of pcDNA, pcDNA-PrP^{C}, pcDNA-PrP^{C} + PLC and pcDNA-PrP^{C} + Gli treated N2a cells. Arrows point proteins of interest (Tubulin and PrP^{C}). sd

Supplementary Figure 3: Full size Western blot corresponding to Figure 4b for Tubulin and PrP^{C} levels in brain extracts obtained from untreated B6129 $Prnp^{Zrchl/Zrchl}$ $Prnp^{+/+}$, B6129 $Prnp^{Zrchl/Zrchl}$ $Prnp^{+/0}$ and B6129 $Prnp^{Zrchl/Zrchl}$ $Prnp^{0/0}$, B6129 $Prnp^{Zrchl/Zrchl}$ $Prnp^{0/0}$ ΔC4 and B6129 $Prnp^{Zrchl/Zrchl}$ $Prnp^{0/0}$ ΔF35 mice. (**a**) 2 minutes exposure. (**b**) 5 minutes exposure. L points to the ladder lines. Notice that the L lines and the lines showing B6129 $Prnp^{Zrchl/Zrchl}$ $Prnp^{+/0}$ were omitted in Fig. 4b. Red arrows points the portions of the Western blot showed in the Figure 4b.

Supplementary Figure 4: Neuronal death in ΔF35 and ΔC4 mice. Low power photomicrographs of Nissl-stained sagittal sections of the hippocampus (**a,b**) and cerebellum (**c-f**) of ΔF35 (**a,c,e**) and ΔC4 (**b,d,f**) mice at 90 days. Note that the hippocampus (**a,b**) of both mutants remains healthy with a well defined pyramidal layer. In contrast, ΔF35 mice showed relevant cerebellar degeneration starting in the granule cell layer in medial cerebellar folia (box in **c**). A higher magnification of the box in (**c**) is showed in (**e**). Arrows in (**e**) point to Purkinje cells and cerebellar folia numbering is included in (**c** and **d**)**.** Abbreviations as in Figure 2 and ml = molecular layer; pcl = pukinge cell layer and gl = granule cell layer in (**e** and **f**). Scale bars: **a** = 200 μm pertains to **b-d; e** = 100 μm pertains to **f**.

Supplementary Figure 5: Full size Western blot corresponding to Figure 5b for Tubulin and PrP^{C} levels in protein extracts of pcDNA, pcDNA-PrP^{C}, pcDNA-$PrP^{\Delta CD}$ and pcDNA-$PrP^{\Delta F35}$ treated N2a cells. (**a**) 2 minutes exposure. (**b**) 5 minutes exposure. Red arrows points the parts of the Western blot showed in the Figure 5b.

Supplementary Movie 1. Representative examples of KA-induced seizures in B6129 and B6.129 *Prnp*$^{0/0}$ mice*.* Results from the microsatellite analysis are included in the first frames of the video in the bottom right corner. The genotype of each mouse is also showed.

Supplementary Movie 2. Three examples of KA-induced seizures in 129/Ola$^{Edbg/Edbg}$ *Prnp*$^{0/0}$ mice*.* Results from the microsatellite analysis are included in the first frames of the video as in Supplementary Movie 1. The genotype of each mouse is also included in the video.

Supplementary Movie 3. Examples of KA-induced seizures in FVB/N *Prnp*$^{0/0}$ *and Prnp*$^{+/+}$ mice*.* The genotype of each mouse is also is showed in the video.

Supplementary Movie 4. Examples of KA-induced seizures in ΔF35 and ΔC4 mice (B6129 *Prnp*$^{0/0}$ background)*.* The genotype of each mouse is showed in the video.

Animal Code	Seizure Intensity Reached			
	Grade I-IV	Grade V	Grade VI	Death
B6129.Ko.1				
B6129.Ko.2				
B6129.Ko.3				
B6129.Ko.4				
B6129.Ko.5				
B6129.Ko.6				
B6129.Ko.7				
B6129.Ko.8				
B6129.Ko.9				
B6129.Ko.10				
B6129.Ko.11				
B6129.Ko.12				
B6129.Ko.13				
B6129.Ko.14				
B6129.Ko.15				
B6129.Ko.16				
B6129.Ko.17				
B6129.Ko.18				
B6129.Ko.19				
B6129.Ko.20				

Animal Code	Seizure Intensity Reached			
	Grade I-IV	Grade V	Grade VI	Death
B6129.Wt.1				
B6129.Wt.2				
B6129.Wt.3				
B6129.Wt.4				
B6129.Wt.5				
B6129.Wt.6				
B6129.Wt.7				
B6129.Wt.8				
B6129.Wt.9				
B6129.Wt.10				
B6129.Wt.11				
B6129.Wt.12				
B6129.Wt.13				
B6129.Wt.14				
B6129.Wt.15				
B6129.Wt.16				

Animal Code	Seizure Intensity Reached			
	Grade I-IV	Grade V	Grade VI	Death
129/Ola.Ko.1				
129/Ola.Ko.2				
129/Ola.Ko.3				
129/Ola.Ko.4				
129/Ola.Ko.5				
129/Ola.Ko.6				
129/Ola.Ko.7				
129/Ola.Ko.8				
129/Ola.Ko.9				

Animal Code	Seizure Intensity Reached			
	Grade I-IV	Grade V	Grade VI	Death
129/Ola.Wt.1				
129/Ola.Wt.2				
129/Ola.Wt.3				
129/Ola.Wt.4				
129/Ola.Wt.5				
129/Ola.Wt.6				
129/Ola.Wt.7				
129/Ola.Wt.8				
129/Ola.Wt.9				
129/Ola.Wt.10				
129/Ola.Wt.11				

Animal Code	Seizure Intensity Reached			
	Grade I-IV	Grade V	Grade VI	Death
FvB/N.Ko.1				
FvB/N.Ko.2				
FvB/N.Ko.3				
FvB/N.Ko.4				
FvB/N.Ko.5				
FvB/N.Ko.6				
FvB/N.Ko.7				

Animal Code	Seizure Intensity Reached			
	Grade I-IV	Grade V	Grade VI	Death
FvB/N.Wt.1				
FvB/N.Wt.2				
FvB/N.Wt.3				
FvB/N.Wt.4				
FvB/N.Wt.5				
FvB/N.Wt.6				
FvB/N.Wt.7				
FvB/N.Wt.8				

a

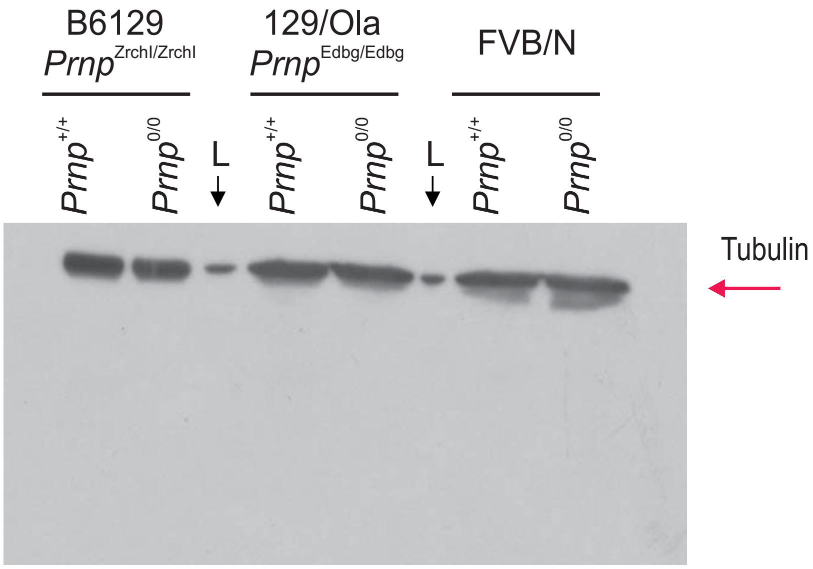

Tubulin Figure 1b

b

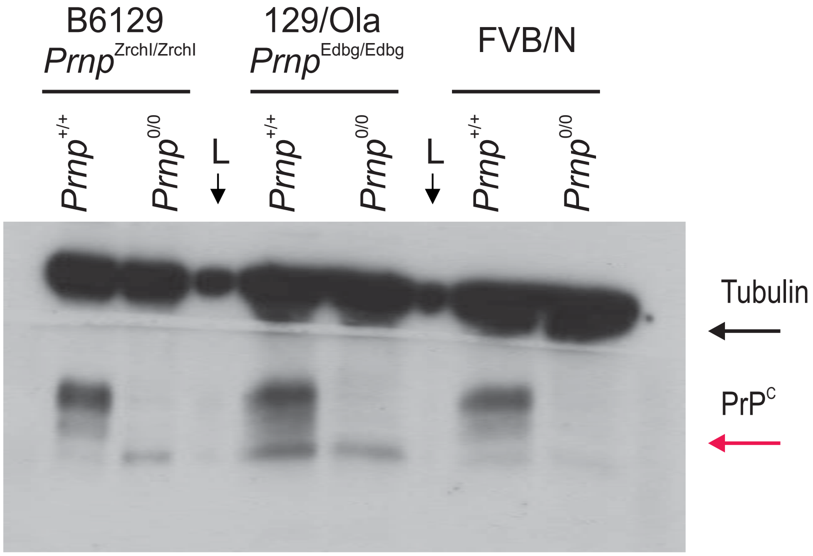

PrP^{C} Figure 1b

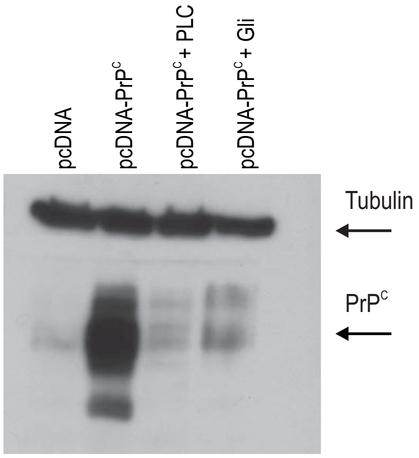

Tubulin and PrP^C Figure 3a

a

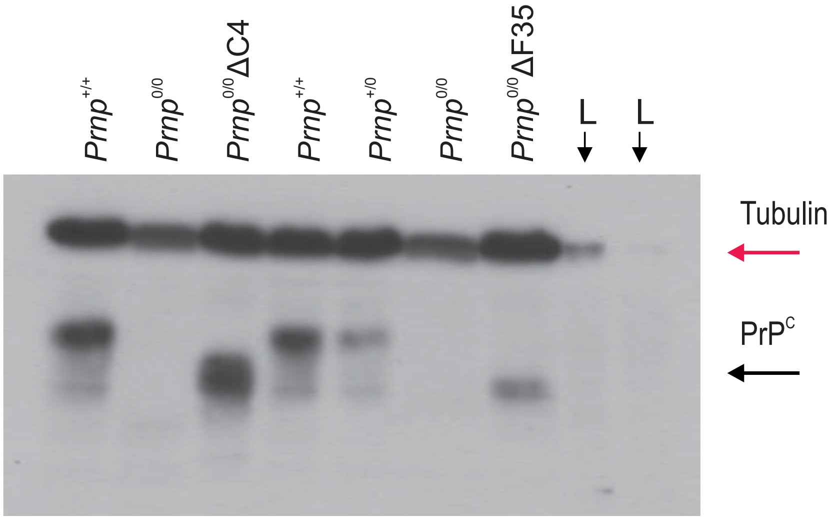

Tubulin Figure 4b

b

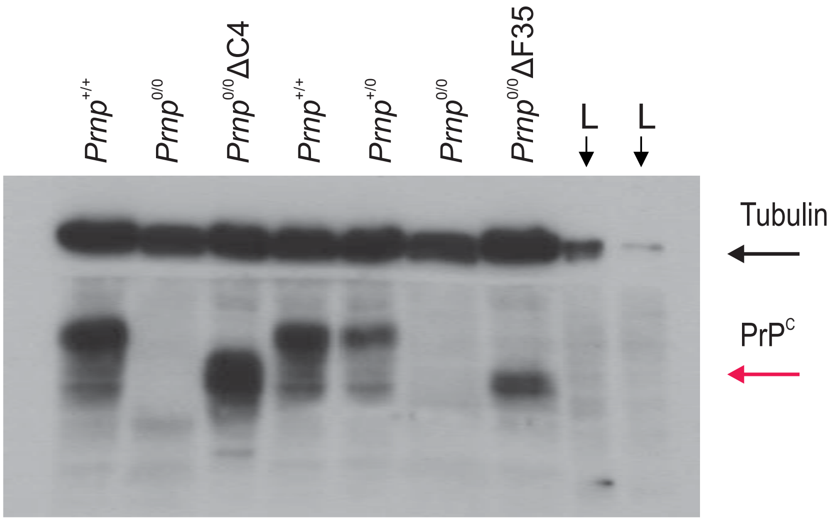

PrP^C Figure 4b

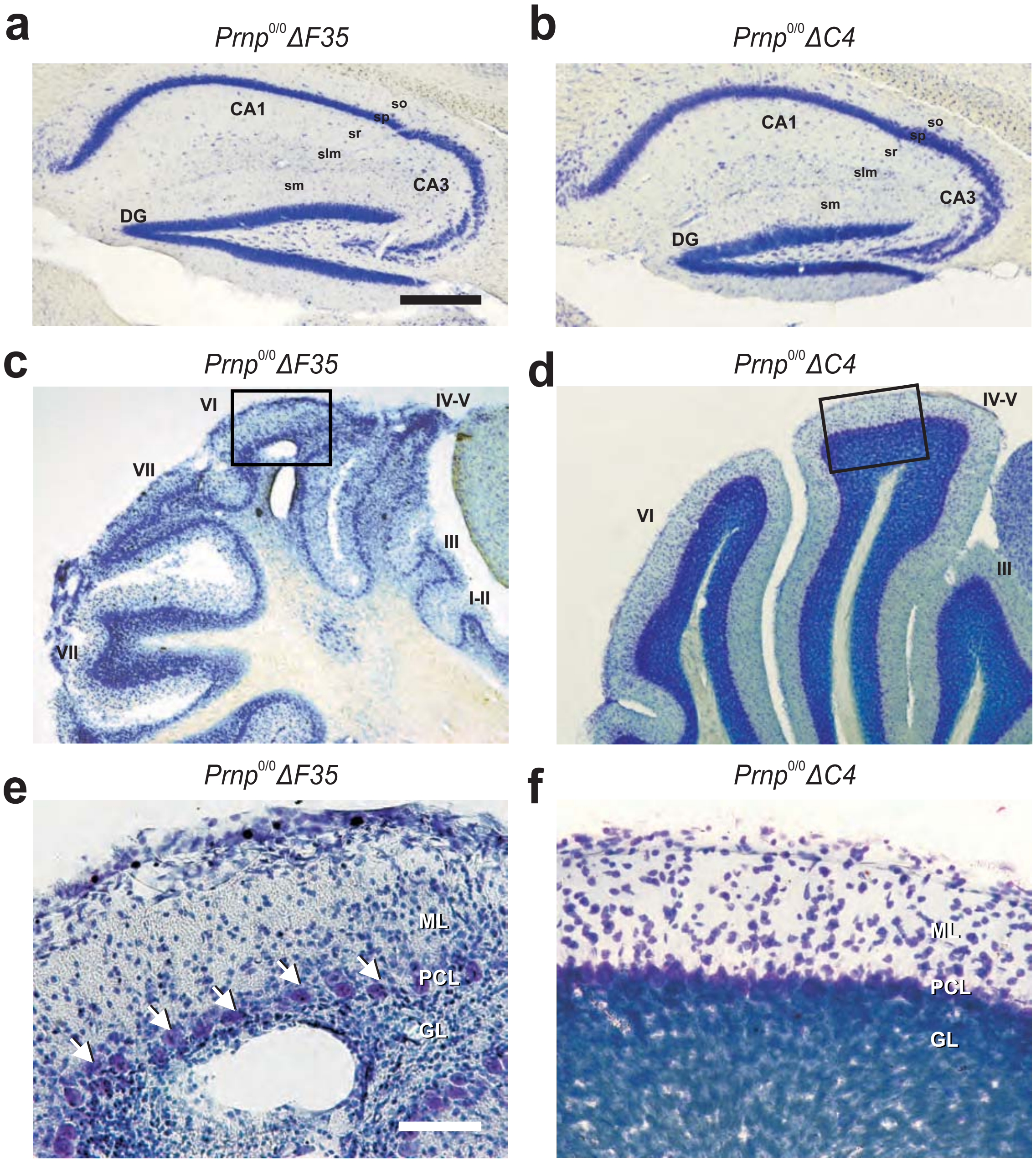
a
Prnp0/0ΔF35
CA1
so
sp
sr
slm
sm
CA3
DG
b
Prnp0/0ΔC4
CA1
so
sp
sr
slm
sm
CA3
DG
c
Prnp0/0ΔF35
VI
IV-V
VII
III
I-II
VII
d
Prnp0/0ΔC4
IV-V
VI
III
e
Prnp0/0ΔF35
ML
PCL
GL
f
Prnp0/0ΔC4
ML
PCL
GL

a

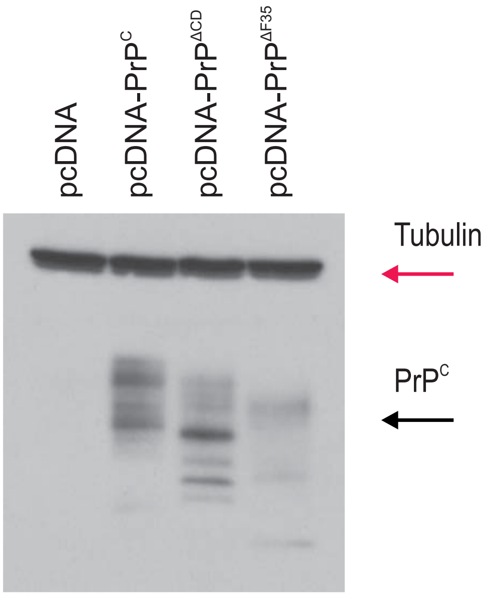

Tubulin Figure 5b

b

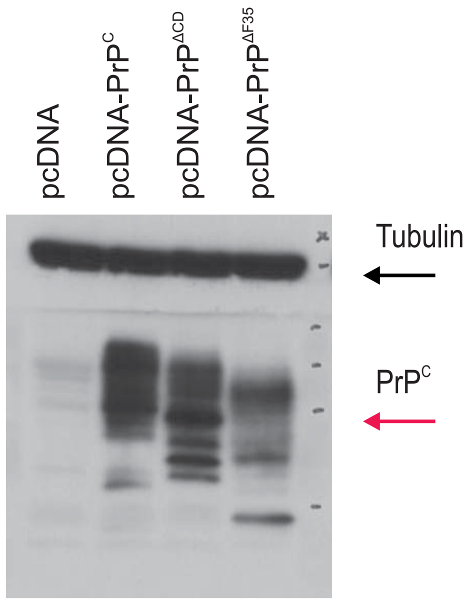

PrP^C Figure 5b
